# The Development of a Multidimensional Inventory for the Assessment of Mental Pain (FESSTE 30)

**DOI:** 10.3389/fpsyg.2021.656862

**Published:** 2021-04-28

**Authors:** Karin Flenreiss-Frankl, Jürgen Fuchshuber, Human Friedrich Unterrainer

**Affiliations:** ^1^Department of Social Sciences, University of Nicosia, Nicosia, Cyprus; ^2^Center for Integrative Addiction Research (CIAR), Grüner Kreis Society, Vienna, Austria; ^3^Department of Religious Studies, University of Vienna, Vienna, Austria; ^4^University Clinic for Psychiatry and Psychotherapeutic Medicine, Medical University Graz, Graz, Austria

**Keywords:** trauma, mental pain, questionnaire development, validation, factor analysis

## Abstract

**Background:** Although the term “mental pain” is often the subject of expert opinions regarding claims for damages, there is still no standardized questionnaire in the German-speaking area to operationalize this concept. Therefore, the aim of this work is the development and validation of a self-assessment measurement for psychological pain after traumatic events (FESSTE).

**Methods:** *A first version of the questionnaire was applied on a sample of the German speaking general population (N* = *425; 88% female). After performing an item analysis and exploratory factor analysis, the questionnaire was shortened and tested on a second German speaking general population sample (N* = *619; 89% female)*. Finally, the newly developed questionnaire was related to the extent of traumatization (measured with a uniquely designed trauma checklist attached to the FESSTE) and already established instruments for the assessment of psychiatric symptom burden, which included the Brief Symptom Inventory-18 (BSI-18) and the Post-traumatic-Stress-Scale (PTSS-10).

**Results:** The final version of the FESSTE consists of a total of 30 items and covers the subscales “Somatization,” “Depression,” “Intrusive Memories,” “Dissociation” and “Anxiety,” and a total scale “Mental Pain.” Based on the confirmatory factor analysis, it is assumed that the latent factor structure of the FESSTE can be best described as a bifactor-model. The final version shows a satisfactory model fit, high internal consistencies, and strong positive correlations with the BSI-18 and PTSS-10, as well as the extent of traumatic experiences.

**Discussion:** The FESSTE enables an operationalization of mental pain comprising five subscales and one total scale. What is more, the trauma checklist attached to the FESSTE allows for the standardized assessment of potentially traumatic experiences and the corresponding extent of these experiences. The results indicate that the FESSTE is a reliable and valid self-assessment procedure for mental pain, which is suitable for use in research and in expert practice.

## Introduction

The Austrian judicative is increasingly aware of the phenomenon of mental pain. In correspondence to this, mental pain is often framed as a psychotraumatic state of suffering with the extent of a disease (Barolin et al., 1994). Psychosomatic as well as psychopathological suffering caused by bodily harm—even without demonstrable physical injury consequences—justify a claim to pain compensation if the psychological impairment is deemed to require treatment or at least can be diagnosed by a doctor. However, mere agitation or outrage about damage, does not qualify as mental pain (Danzl et al., 2019).

Based on Holczabek's (1976) classification of physical pain, which is commonly referenced in Austrian legal opinions regarding the assessment of compensations, Laubichler (2002) defined three categories of mental pain: (1) Mild mental pain which is distinguished by a slight impairment of the ability to work, since mild mental pain only occurs intermittently and incidentally. This means, for example, that depression, anxiety and fears can be adequately dealt with by the affected subject. (2) Moderate pain is characterized by a balance of the ability to carry out activities in a professional or other sense and the inability to do so. Hence, moderate mental pain implies that activities are possible but significantly impaired. (3) Finally, in the case of severe mental pain, the subject is helpless against the pain and unable to detach himself from it. Therefore, the implementation of useful activities is inhibited, as the mental pain is so paramount that they occupy the entire space of consciousness.

Historically, there have been several attempts to define mental pain. From a psychoanalytic perspective, Freud's “Studies on Hysteria” already discussed the phenomena of mental pain (Freud and Breuer, 1895/2000). In this work he related the avoidance of the perception of mental pain caused by traumatic events to the development of hysterical symptoms. In his later writings “Mourning and Melancholia” (Freud, 1924), as well as “Inhibitions, Symptoms and Anxiety” (Freud, 1926/1975), he attributes mental pain to the specific feelings of sadness after traumatic experiences of loss. According to Freud (1924, 1926/1975) mental pain is functionally in the service of the detachment of libidinal energy from the lost love object. He sees this in contrast to physical pain, which in his view is characterized by a deduction of narcissistic libido from his own body parts.

Later, Bakan (1968) similarly emphasized the important role of social loss experiences in the development of mental pain. According to its definition, mental pain denotes the awareness of a disturbance in the human tendency toward a state of wholeness and social well-being. Furthermore, Frankl (1963) associates psychological pain primarily with the feeling of an agonizing inner emptiness. For him, this is the result of a loss of meaningfulness in one's own life and can therefore only be modulated by adjusting values that enable the individual to give his life a new meaning. Another definition can be found in the writings of Sandler (1962) and Joffe and Sandler (1965). In their conceptualization, mental pain is the result of a discrepancy between the ideal self and the perception of the real self. These self-representations are composed of mental images that the individual has of oneself and one's social role.

Moreover, Baumeister's (1990) theory on suicide as an escape from oneself poses a similar approach. For him, mental pain is caused by a strongly pronounced self-perception of inadequacies, which in turn results from negative self-attributions with regard to one's own failures. Specifically, this means that if the ideal self and the actual perception of the actual self are far apart and this difference is ascribed to one's own failures. The subject perceives this as mental pain. Hence, for Baumeister, the underlying effect of mental pain is disappointment in oneself. This contrasts with the Herman's (1992) and Janoff-Bulman's (1992) conceptualization, who define mental pain as the perception of negative changes within the self. Similar to Freud (1926/1975, 1966) and Bakan (1968) they assume that these are essentially related to the experience of trauma and loss of attachment figures.

In the course of his work on suicidality, Shneidman (1977, 1993, 1998) dealt extensively with the phenomenon of mental pain, which he calls “psychache.” He assumes that mental pain is fed by the frustration of essential needs of the individual, such as being loved, need for control and security, protection of self-image, avoidance of shame and the need for understanding. If these basic needs are not sufficiently met, a mixture of different negative emotional states such as guilt, shame, loss, despair, loneliness, grief, hopelessness, and anger occurs (Shneidman, 1993). In this context, Shneidman emphasizes that this variety of negative emotions can lead to a generalized and almost unbearable psychological pain. If this pain reaches an intensity which is too high and it is not possible to predict any positive change for the individual in the future, it often necessitates an attempt to evade this state through suicide.

Bion (1970/2013) introduced a differentiation between the concept of mental pain and suffering. He argued that the emergence of mental pain is linked to traumatic experiences described as “beta elements.” These beta elements are experienced as overwhelming and are not accessible for the patient's “alpha function.” Therefore, they fail to be contained and symbolized and are ultimately expressed as mental pain. In contrast, suffering is associated with negative experiences which can be contained within the mental apparatus. Hence, the emotional pain caused by these events can be processed into “alpha elements” which are suitable for mental elaboration and can be further processed by the patient (Bion, 1970/2013; Fleming, 2006).

Against this psychoanalytic background, Shneidman (1999) also made the first attempt at a questionnaire-based operationalization of the mental pain concept with the original *Psychological Pain Assessment Scale* (PPAS; Shneidman, 1999), which has never been empirically validated. However, on the basis of Shneidman's work, the *Psychache Scale* (Holden et al., 2001) was developed which assesses the intensity of mental pain based by means of 13 items, which are answered on a five-point Likert scale. Empirical findings show a high internal consistency of the one-factorial scale and confirm the construct validity of Shneidman's concept, particularly with regard to the assumed connections with depression, hopelessness, psychiatric symptoms, and increased suicidality (Holden et al., 2001; Mills et al., 2005). However, a German version of this questionnaire is not yet available.

Another approach to research into psychological pain was developed by Bolger (1999). Her definition takes as a starting point a qualitative analysis of narrative descriptions of mental pain in traumatized individuals. Based on these evaluations, she understands mental pain as a torn self (“brokenness of the self”). This conflict is made up of various determinants and includes a feeling of hurt, separation from loved ones, loss of self-esteem, loss of control, and fear. However, Bolger did not develop a standardized measuring instrument for the operationalization of mental pain, as well.

Also on the basis of a qualitative content analysis, but of narratives from psychiatric inpatients and randomly selected healthy test persons (see Orbach and Mikulincer, 2002), Orbach et al. (2003b) developed a questionnaire for the assessment of mental pain. In correspondence to this, Orbach et al. (2003a,b) define mental pain as a broad spectrum of subjective perception of negative changes within the self and its functions. This perception is accompanied by intense negative feelings and is therefore often experienced as torture. The *Orbach and Mikulincer Mental Pain Scale* (OMMP; Orbach et al., 2003b) has nine factors that are operationalized with 44 items that are answered on a five-point Likert scale. The operationalized scales are detailed as: *Irreversibility, Loss of Control, Narcissist Wounds, Emotional Flooding, Freezing, Self-estrangement, Confusion, Social Distancing*, and *Emptiness*. In its validation study the OMMP showed satisfying psychometric criteria (Orbach et al., 2003b). In addition, the results of Orbach et al. (2003a) suggest significant correlations between OMMP and increased suicidality and hopelessness, which is in line with the conceptual framework by Frankl (1963), Baumeister (1990), and Shneidman (1998). These relationships were also highlighted in a recently published systematic review by Verrocchio et al. (2016). Again, no translation has been carried out for the German-speaking area for the OMMP.

In the legal and expert context, mental pain is generally defined as “unbill” or “hardship,” which means as an immaterial damage, which justifies corresponding compensation (Barolin et al., 1994). In expert practice, mental pain is often equated with mental illness. However, the diagnosis of post-traumatic stress disorder (PTSD) as defined by ICD-10 (World Health Organization, 1992) is not a prerequisite for classifying mental pain. It is assumed that, in addition to PTSD, symptoms such as a stress reaction, depression, anxiety disorder or adjustment disorder also cause mental pain. The presence of a mental pain or a psychotraumatic state of suffering of disease value should only be assumed, however, if there is a traumatic event, which must have a clearly demonstrable connection with the psychological state (Barolin et al., 1994).

### Construction of the First Version of the Questionnaire

As a first step in the present study, a qualitative content analysis (Mayring, 2010) of 18 court opinions was carried out. Based on this the scales for the first empirical examination of the FESSTE were determined. The analysis resulted in 28 categories, which were grouped into the superordinate categories “Vegetative Symptoms/Somatization,” “Treatment,” “Compensation Behavior,” “Vulnerability,” “Anxiety Symptoms,” “Trauma Disorder,” “Depression,” and “Mood Disorder.” These categories were used in the next step as starting points for the construction of the individual scales. Due to the lack of meaningfulness, the categories “vulnerability” and “mental disorders” were excluded from the further scale construction. The category “treatment” is covered descriptively in the questionnaire through several separate questions but was not included in the calculation of the mental pain total value.

For the remaining categories—depending on the scope of the theoretical concepts—between 3 and 23 items were formulated. The selection and formulation of the items was based on the developed categories, the ICD-10 (World Health Organization, 1992) and on already established questionnaires for the individual concepts, such as the Symptom-Check-List-90 (SCL-90; Derogatis, 1975), and the Essener Trauma-Inventar (ETI; Tagay et al., 2007). In addition, the items were formulated in the form of statements. Particular emphasis was placed on adhering to specific formulation rules in order to increase comprehensibility and subsequently, the measurement accuracy (Bühner, 2011). These rules include avoiding double negations, using short, unambiguous sentences and avoiding foreign words or technical terms.

On the basis of empirical results that suggest that scaling with a response format of five to seven levels maximizes the reliability of the measuring instrument (Bühner, 2011) a five-point Likert scale was used to assess the individual items. “Not at all (0)—very strong (4)” was chosen as the response format.

Moreover, at the beginning of the questionnaire, a list of potentially traumatic events is given, which is based on the results of epidemiological data from Perkonigg et al. (2000) and the recent trauma definition of the DSM-5 (American Psychiatric Association, 2013), encompassing events connected to actual or menaced death, serious personal injury or sexual violence. The individual items can be answered with “yes” or “no” with regard to the question of whether they happened to the test participant. The category “Yes” is divided into the subcategories “Personal” and “As a witness.” The given answers are evaluated with specific point values (“yes—personal” = 2; “yes—as a witness” = 1; no = 0), which can be summed up to assess the parameter “extent of traumatic experience.”

## Design and Methods

### Sample and Procedure

The sample from the general population examined in the trial phase consisted of 425 German-speaking participants (88% female; age: 18–76 years). In the validation phase, a sample of 619 participants (90% female; age: 18–72 years) was examined. The participants were recruited through advertisements in public forums and social networks (e.g., Facebook). The data was collected via the online study platform LimeSurvey. Informed consent was obtained from all subjects before they filled in the questionnaires. The survey consisted of various demographic questions (e.g., gender, age, education, and psychiatric diagnoses) as well as the standardized test procedures described below. The participants did not receive any compensation. The sole inclusion criterion for participating in the online survey was an age over 18. Participants remained completely anonymous during and after the period of study participation. The study was carried out in accordance with the Helsinki Declaration.

### Measurement Dimensions

#### Trial Phase

A total of 77 items were formulated to cover each of the five initial categories “Vegetative Symptoms / Somatization,” “Compensatory Behavior,” “Anxiety Symptoms,” “Trauma Disorder,” and “Depression,” which were obtained from the qualitative content analysis of court reports.

The queried list of potentially traumatic experiences includes in detail:

(1) Torture; (2) Stay in war zone; (3) Serious accident, fire, or explosion; (4) Natural disaster; (5) Serious illness or injury; (6) Sudden or unexpected death of a close relative or an important person; (7) Displacement and Migration; (8) Imprisonment; (9) Neglect; (10) Sexual violence by a stranger as an adult; (11) Sexual violence by a person from the family or circle of acquaintances; (12) Sexual violence as a child or adolescent by a stranger; (13) Sexual violence as a child or adolescent by a person from the family or circle of friends; (14) Violent attack by a stranger; (15) Violent attack by a person belonging to the family or circle of acquaintances. Furthermore, “Other stressful life events” were assessed as a descriptive dimension which was excluded from the “extent of traumatic experience” score.

#### Psychometric Assessment

In order to evaluate aspects of convergent validity, the following psychometric measurement tools were included in the trial phase:

*Brief Symptom Inventory* (BSI-18; Derogatis, 2001; German version: Spitzer et al., 2011). The BSI-18 is a short version of the SCL-90-R (Derogatis, 1975). This self-report measurement is used to assess psychological distress within the last 7 days and consists of 18 items. The questionnaire includes the subscales “Depression,” “Anxiety,” and “Somatization,” as well as an overall scale for assessing the general burden of symptoms, which is called “Global Severity Index” (GSI). The individual items are answered using a five-point Likert scale, which ranges from 0 (“not at all”) to 4 (“very much”). Reliability for the BSI-18 ranged from excellent to acceptable with Cronbach's α = 0.93 (GSI) – 0.79 (Somatization).

The *Post-traumatische-Stress-Scale-10* (PTSS-10; Holen et al., 1990; German version: Maercker, 2003), is a screening instrument for the assessment of PTSD symptoms. It comprises 10 items, which are answered in the current version on a seven-point Likert scale (from 0 = “never” to 6 = “always”). Subjects were asked about the extent of typical PTSD symptoms within the last week. The questionnaire covers the following areas: (1) “Sleep Problems”; (2) “Nightmares”; (3) “Depression”; (4) “Jumpiness”; (5) “Withdrawal Tendencies”; (6) “Irritability”; (7) “Mood Swings”; (8) “Feelings of Guilt”; (9) “Anxiety,” and (10) “Muscle Tension.” The reliability of this scale was excellent with Cronbach's α = 0.90 (*N* = 425).

#### Validation Phase

Based on the results of the item characteristic analysis, exploratory factor and reliability analysis, the revised version of the questionnaire was given to a new sample of the general population. For this aim, the questionnaire was shortened to overall 52 items, operationalizing the scales “Somatization” (9 items), “Anxiety” (12 items), “Depression” (14 items), “Intrusive Memories” (11 items), and “Dissociation” (6 items).

#### Statistical Analysis and Analysis Strategy

The statistical analysis was conducted via SPSS 25.0 and AMOS 24.0. SPSS was used for data management and the calculations of descriptive statistics, reliabilities, exploratory factor analysis, bivariate correlations, ANOVAs and multivariate hierarchical regression analysis. The confirmatory factor analysis was estimated with AMOS. The total sample of 1,044 participants is divided into two groups (A = 425; B = 619). In group A, an exploratory factor analysis (varimax rotation, analysis of the 5-factorial solution) and the calculation of aspects of convergent validity were carried out. A confirmatory factor analysis was performed on the data from group B. Descriptive statistics and reliability of the final FESSTE were assessed based on the total sample.

With regard to the confirmatory factor analysis, the following fit indices were accepted as indicators for an acceptable model fit (Kline, 2015): (a) Comparative Fit Index (*CFI*) >0.90; (b) Tucker-Lewis Index (*TLI*) >0.90; (c) Normed Fit Index (NFI) >0.90; (d) Square Root Error of Approximation (*RMSEA*) <0.08 with the upper limit of the 90% confidence interval <1; (e) a χ^2^/*df* <3. The model fit of competing models was assessed by comparing the AIC values. The model with the lowest AIC value was preferred. A ΔAIC >2 was seen as an indication of a significant difference between the models (Cheung and Rensvold, 2002; Jovanović, 2015). In the case of a poor model fit, the items with the lowest power of discrimination with regard to the assigned factors were removed from the model. This process was repeated until the model had a satisfactory fit. In accordance with Kline (2015), all indicators were logarithmically transformed for the confirmatory factor analysis, due to the lack of a normal distribution of the individual items (Kline, 2015).

## Results

### Sample Characteristics

The descriptive sample characteristics for the trial and validation phases are detailed in Table 1. The mean age of the participants of the trial phase (sample A; *N* = 425) and the validation phase (sample B; *N* = 619) was 35 years (A: SD = 13 years; B: SD = 11 years). Three hundred and seventy-four (88%) of the participants in sample A were female, compared to 555 (90%) participants in group B. Three hundred and eighty-one (A: 90%) and 600 (B: 96%), respectively, stated at least one lifetime traumatic experience or stressful life event. Most participants' highest educational qualification was a qualification for higher education (A: *n* = 161; 38%; B: *n* = 185; 30%). Furthermore, the majority was in employment during the time of the study (A: *n* = 210; 50%; B: *n* = 365; 59%). Concerning the relationship status, the greater part of sample A was single (*n* = 156; 32%), which is contrasted by sample B which had a majority of participants in a relationship (*n* = 134; 36%). Regarding nationality most subjects in both groups were Austrian (A: *n* = 353; 83.1%; B: *n* = 532; 86%). A significant proportion of probands reported some form of medication due to health problems related to a traumatic incident (A: *n* = 120; 28%; B: *n* = 228; 37%) Finally, 78 (20%) participants in group A declared to be diagnosed with a psychiatric disorder, compared to 77 (12%) in group B.

**Table 1:** Sample characteristics (testing and validation phase).

**Sample**	**Exploration phase**	**Validation phase**
Overall	*N* = 425	*N* = 619
Gender	*N* = 374 Female (88%)/ *N* = 44 Male (22%)	*N* = 555 Female (90%)/ *N* = 64 Male (10%)
Age	M = 35 (*SD* = 13 Jahre)	M = 35 (*SD* = 11 Jahre)
At least one traumatic life event	*N* = 383 Yes (90%)/ *N* = 42 No (10%)	*N* = 600 Yes (96%)/ *N* = 19 No (4%)
Highest finished education	*N* = 161 General qualification for university entrance (38%)/ *N* = 103 Master University degree (24%)/ *N* = 67 Bachelor University degree (16%)/ *N* = 53 Apprenticeship (12%)/ *N* = 21 Phd (5%)/ *N* = 17 High School (4%)/ *N* = 3 None (1%)	*N* = 185 General qualification for university entrance (30%)/ *N* = 140 Master University degree (22%)/ *N* = 121 Bachelor University degree (19%)/ *N* = 119 Apprenticeship (19%)/ *N* = 10 Phd (2%)/ *N* = 39 High School (6%)/ *N* = 5 None (1%)
Occupation	*N* = 210 in employment (50%)/ *N* = 128 in education (30%)/ *N* = 65 Unemployed (15%)/ *N* = 22 in Pension (5%)	*N* = 365 in employment (59%)/ *N* = 141 in education (22%)/ *N* = 81 Unemployed (13%)/ *N* = 32 in Pension (5%)
Relationship status	*N* = 156 Single (32%)/ *N* = 134 in relationship (32%)/ *N* = 84 Married (20%)/ *N* = 45 Divorced (10%)/ *N* = 6 Widowed (1%)	*N* = 201 Single (32%)/ *N* = 222 in relationship (36%)/ *N* = 118 Married (19%)/ *N* = 69 Divorced (11%)/ *N* = 9 Widowed (1%)
Nationality	*N* = 353 Austrian (83%)/ *N* = 40 German (9%)/ *N* = 32 Other (8%)	*N* = 532 Austrian (86%)/ *N* = 59 German (10%)/ *N* = 28 Other (5%)
Psychiatric diagnosis	*N* = 347 No (81%)/ *N* = 78 Yes (19%)	*N* = 542 No (88%)/ *N* = 77 Yes (12%)

### Descriptive Analysis of the Extent of Traumatization

As detailed in Table 2, the most commonly reported traumatic event was the sudden death of a close relative or an important caregiver (A: *n* = 212; B: *n* = 328). The personal experience of violent attacks by people from family or acquaintances (A: *n* = 107; B: *n* = 208), and serious illness or injury (A: *n* = 105; B: *n* = 191) were also frequently stated. A large number of participants also reported some form of sexual abuse. In this context, “sexual violence by a person from family or friends as a child or adolescent” was mentioned most frequently in both groups (A: *n* = 76; B: *n* = 127). A substantial proportion of participants also reported the experience of other stressful life events (e.g., mobbing, being left by a partner, stalking, and loss of a job) not listed in the questionnaire (A: *n* = 130; B: *n* = 219). Furthermore, results of the reliability analysis regarding the “extent of traumatic experience” total score was within an acceptable range with Cronbach's α = 0.63 (*N* = 1,044).

**Table 2:** Sample characteristics regarding experienced trauma.

**Sample**	**Trial phase (*****N*** **= 381)**				**Validation phase (*****N*** **= 600)**			
**Type of traumatization**	**Personal**		**As witness**		**Personal**		**As witness**	
Torture	11	3%	13	3%	25	4%	11	2%
Stay in war zone	12	3%	9	2%	12	2%	12	2%
Serious accident, fire or explosion	58	15%	61	16%	92	15%	82	14%
Natural disaster	13	3%	28	7%	44	7%	34	6%
Serious illness or injury	105	28%	148	39%	191	32%	187	31%
Sudden/unexpected death of a close relative or an important person	212	56%	54	14%	328	55%	82	14%
Flight and migration	14	4%	42	11%	10	2%	34	6%
Captivity	10	3%	8	2%	11	2%	11	2%
Neglect	67	18%	49	13%	115	19%	51	9%
Sexual violence by a stranger as an adult	50	13%	10	3%	91	15%	22	4%
Sexual violence by a person from the family or circle of acquaintances as an adult	66	17%	16	4%	116	19%	13	2%
Sexual violence by a stranger as a child or adolescent	51	13%	9	2%	86	14%	10	2%
Sexual violence by a person from the family or circle of acquaintances as a child or adolescent	75	20%	14	4%	138	23%	14	2%
Violent attack by a stranger	76	20%	55	14%	127	21%	60	10%
Violent attack by a member of the family or circle of acquaintances	107	28%	24	6%	208	35%	36	6%
Other traumatic events	130	34%	14	4%	213	36%	12	2%

### Exploratory Analysis of the FESSTE Factor Structure

The exploratory factor analysis was carried out on the basis of the main component analysis with a VARIMAX rotation and was a priori set to five factors. The five factors solution explained 56.96% of the total variance. In the rotated factor solution, the first factor “depressive symptoms” was comprised of 14 items with factor loadings ranging from 0.78 to 0.42, showed an eigenvalue of 8.43 and explained 16.20% of the variance. The further course of the components can be given with eigenvalues of 6.19 (11.90%) for “Dissociation” which included six items (factor loadings: 0.72 – 0.44); “Fear” which showed an eigenvalue of 5.61 with 10.79% explained variance and was comprised of 12 items with factor loadings ranging 0.77 – 0.45; “Intrusive Memories” exhibiting an eigenvalue of 5.47 with 10.52% explained variance and 11 Items (factor loadings ranging from 0.72 to 0.49); and finally, “Somatization” which had an eigenvalue of 3.92 with 7.54% explained variance and 9 items ranging from 0.70 to 0.46 regarding their factor loadings.

### Analysis of the Latent Factor Structure of the FESSTE

As shown in Table 3, no model of the original 52-item version of the FESSTE showed a satisfactory fit (all models: CFI <0.90; TLI <0.90; NFI <0.90; χ^2^/*df* > 3). Subsequently, the number of items for the respective factors was step wise reduced, in consideration of the selectivity of the separate items. Overall, this approach led to a substantial increase in the model fit. In the 30-item version, the individual subscales of the FESSTE showed a generally acceptable model fit. The most promising parameters were achieved with the bifactor model of the 30-item version of the FESSTE. In this model, all global fit indices were satisfactory: RMSEA = 0.05 (90% CI: 0.05, 0.06); TLI = 0.92; CFI = 0.93; NFI = 0.90; AIC = 1211.03.

**Table 3:** Results of the confirmatory factor analysis of several FESSTE models.

**Modell**	**χ^2^(*df*)**	**χ^2^/*df***	**RMSEA (90% CI)**	**CFI**	**NFI**	**TLI**	**AIC**
**52-Item Version**							
One-factor	8737.39 (1,280)	6.83	0.098 (0.096–0.100)	0.58	0.54	0.56	8933.39
Five-factor	4897.27 (1,264)	3.87	0.069 (0.067–0.071)	0.80	0.74	0.76	4897.27
One-factor of higher order	4948.04 (1,269)	3.90	0.069 (0.067–0.071)	0.79	0.74	0.78	5166.04
Bifactor	4656.32 (1,228)	3.79	0.069 (0.066–0.070)	0.80	0.76	0.79	4956.32
**30-Item Version**							
One-factor	3465.33 (405)	8.56	0.112 (0.108–0.115)	0.68	0.65	0.65	3465.33
Five-factor	1331.76 (395)	3.37	0.063 (0.059–0.066)	0.90	0.87	0.89	1471.76
One-factor of higher order	1359.48 (400)	3.40	0.063 (0.059–0.067)	0.90	0.86	0.89	1489.49
Bifactor	1031.03 (375)	2.75	0.054 (0.050–0.058)	0.93	0.90	0.92	1211.03

*N = 619*.

**Figure 1:**
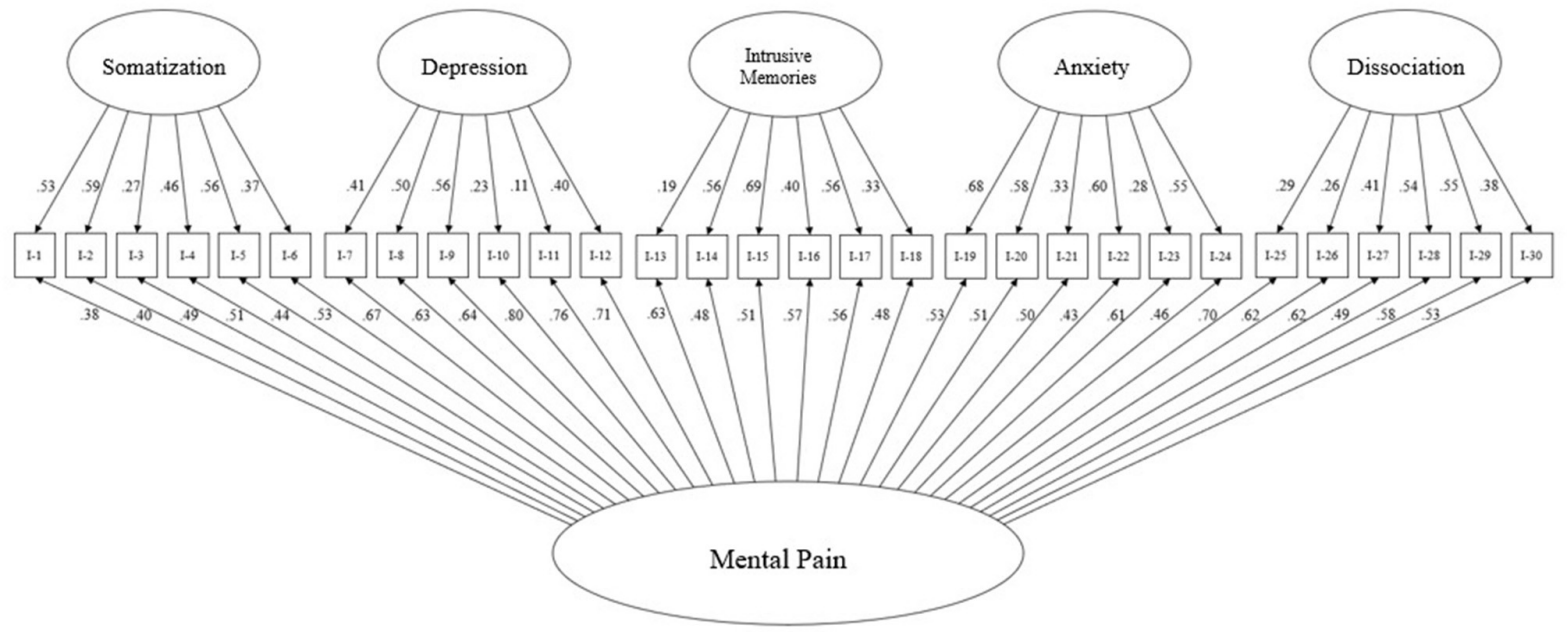
Bifactor model of the 30-item version of the Questionnaire for the Assessment of Mental Pain after Traumatic Events (FESSTE 30) (*N* = 619).

As shown in Figure 1, this model showed a strong overall factor “mental pain,” which is significantly associated with all items (β = 0.38–0.80; all *p* < 0.001). Overall, the loadings of the sub-factors on the respective items were somewhat less pronounced. Regarding the subscales, “fear” showed significant associations in the range between β = 0.28–0.68 (all *p* < 0.001). The regression coefficients on the scale “Intrusive Memories” scale ranged between β = 0.19–0.69 (all *p* < 0.001). “Depression” was associated with the assigned items within the range β = 0.11–0.50 (p <0.01–0.001). The associations between the items and the latent factor “dissociation” were between β = 0.29–0.55 (all *p* < 0.001). Finally, the “somatization” scale showed associations in the range β = 0.27–0.56 (all *p* < 0.001).

### Correlation With Psychiatric Symptoms and Psychometric Properties of the FESSTE

As can be seen in Table 4, the overall scale showed excellent internal consistency (Cronbach's α = 0.95). Furthermore, the subscales also showed satisfactory internal consistencies (Cronbach's α = 0.82–0.91). Table 5 also details the correlations of the subscales with the overall scale and the intercorrelations between the subscales. With regard to the correlation patterns, it should be noted that the subscales of the FESSTE were intercorrelated less with one another than with the overall scale value, which can be interpreted as a preliminary confirmation of the derived factor structure.

**Table 4:** Correlation between the FESSTE scales and indicators of symptom severity.

**Variable**	**FESSTE 30 total score**	**FESSTE 30 somatization**	**FESSTE 30 depression**	**FESSTE 30 anxiety**	**FESSTE 30 dissociation**	**FESSTE 30 intrusive memories**
Extent of traumatic experiences	0.52^*^	0.48^*^	0.42^*^	0.42^*^	0.45^*^	0.42^*^
BSI depressiveness	0.78^*^	0.50^*^	0.90^*^	0.55^*^	0.65^*^	0.58^*^
BSI anxiety	0.83^*^	0.63^*^	0.72^*^	0.79^*^	0.63^*^	0.68^*^
BSI somatization	0.73^*^	0.80^*^	0.54^*^	0.59^*^	0.52^*^	0.61^*^
GSI (BSI-18)	0.91^*^	0.72^*^	0.86^*^	0.74^*^	0.70^*^	0.72^*^
PTSS-10	0.88^*^	0.67^*^	0.81^*^	0.70^*^	0.69^*^	0.75^*^

*N = 425*.

* *p < 0.001; GSI = BSI-18 total score*.

**Table 5:** Internal consistency and intercorrelation of the FESSTE 30 scales.

**Variable**	**1**	**2**	**3**	**4**	**5**	**6**
1. Somatization	–					
2. Intrusive memories	0.52^*^	–				
3. Anxiety	0.53^*^	0.58^*^	–			
4. Depression	0.52^*^	0.62^*^	0.55^*^	–		
5. Dissociation	0.47^*^	0.57^*^	0.54^*^	0.70^*^	–	
6. FESSTE total score	0.74^*^	0.83^*^	0.78^*^	0.86^*^	0.81^*^	–
Cronbach's α	0.82	0.86	0.87	0.91	0.86	0.95

*N = 1,044;*

* *p < 0.001; M, mean; SD, standard deviation*.

As shown in Table 5, the subscales and the overall scale of the FESSTE show medium to high positive correlations with the external criteria (*r* = 0.50 −0.91; all *p* < 0.001) and with the reported extent of experienced traumatic experiences (*r* = 0.42 −0.52; all *p* < 0.001; see Cohen, 1992). Strong correlations were observed between the FESSTE total score and the BSI-18 (*r* = 0.91; *p* < 0.001) and the PTSS-10 (*r* = 0.88; *p* < 0.001).

Regarding the examination of the distribution characteristics which was based on the Kolmogorov-Smirnov adaptation test for normal distribution and the analysis of the skewness and kurtosis, it can be assumed that none of the scales exhibited a normal distribution (see Table 6). In a sample of the general population, the subscales and the overall scale show a clearly skewed right-hand distribution. With regard to kurtosis, the scales “Somatization,” “Intrusive Memories,” “Depression,” and the overall scale show a flattened distribution, while the scales “Fear” and “Dissociation” show a sharp distribution.

**Table 6:** Questionnaire for the Assessment of Mental Pain after Traumatic Events (FESSTE 30): mean, standard deviation, and distribution characteristics.

							Test for normal distribution^*^	
**Variable**	**M**	**SD**	**Min**	**Max**	**Skew**	**Kurtosis**	***z***	***p***
1. Somatization	6.47	5.42	0	24	0.84	−0.08	4.23	0.00
2. Intrusive memories	9.02	6.76	0	24	0.46	−0.82	3.28	0.00
3. Anxiety	4.85	5.58	0	24	1.38	1.19	6.22	0.00
4. Depression	8.87	6.98	0	24	0.48	−0.90	3.78	0.00
5. Dissociation	5.71	5.79	0	24	1.04	0.27	5.24	0.00
6. FESSTE total score	34.94	24.67	0	114	0.54	−0.47	2.75	0.00

*N = 1,044; M, mean; SD, standard deviation*.

* *Kolmogorov-Smirnov-test*.

### Influence of Age and Gender on Mental Pain

With regard to age effects on the FESSTE scales, small negative correlations between age and the subscales “trauma-related disorder” (*r* = −0.07; *p* < 0.05), “anxiety” (*r* = −0.06; *p* < 0.05) and dissociation (*r* = −0.10; *p* < 0.05) were observed. Moreover, there is a small positive correlation between age and the extent of traumatic experiences (*r* = −0.07; *p* < 0.05). No significant correlations were found between the age of the subjects and the FESSTE total scale (*r* = −0.04; *p* = 0.21) and the Subscale “Depressive Symptoms” (*r* = −0.04; *p* = 0.17).

In the overall scale and all subscales, female subjects showed significantly higher values (*F* = 22.96–57.63; all *p* < 0.001; η^2^ = 0.02–0.05). Women also reported a slightly higher extent of traumatic experiences (*F* = 5.36; *p* < 0.05; η^2^ = 0.01).

### Comparison of the Prediction of the Extent of Traumatic Experiences Between the FESSTE, BSI-18, and PTSS-10

A comparison regarding the prediction of the extent of traumatic experiences between the established measuring instruments PTSS-10 and BSI-10, as well as the newly developed FESSTE was carried out with a multivariate hierarchical regression analysis. For this aim, sex and age was entered as a control variable at step 1 (*R*^2^ = 0.04; ΔF = 8.93; *p* < 0.001). In step 2 the PTSS-10 total score and the BSI-18 total score were added (*R*^2^ = 0.25; ΔF = 60.25; *p* < 0.001). Finally, step 3 included the FESSTE total score (*R*^2^ = 0.30; ΔF = 28.75; *p* < 0.001). The results suggested that the FESSTE enables a significantly higher explanation of variance than the two comparison instruments (ΔR = 0.05; β = 0.59; *p* < 0.001; see Table 7).

**Table 7:** Hierarchical multiple regression to predict the extent of traumatic experience controlled for gender and age.

	**Variable**	***R*^**2**^**	**ΔR^**2**^**	**ΔF**	**β**
Step 1		0.04	0.04	8.93^***^	
	Gender				−0.18^***^
	Age				0.12^*^
Step 2		0.25	0.21	60.25^***^	
	Gender				−0.04
	Alter				0.14^**^
	PTSS-10				0.33^***^
	GSI				0.17^*^
Step 3		0.30	0.05	28.75^***^	
	Gender				−0.05
	Age				0.15^***^
	PTSS-10				0.07
	GSI				0.09
	FESSTE Total score				0.59^***^

*N = 425; Criteria = Extent of traumatic experience; Gender was coded as: Female = 0; Male = 1; GSI = BSI-18 total score;*

*** *p < 0.001;*

** *p < 0.01; and*

* *p < 0.05*.

## Discussion

The aim of the present work was to develop and validate a self-report measurement for the operationalization of mental pain, since this concept is of great importance in expert practice (Laubichler, 2002), yet to date no standardized questionnaires for the German speaking area exists which operationalizes this construct.

On the basis of qualitative evaluations of judicial reports, 8 factors were originally assumed, which could be reduced to an overall factor and five sub-factors in the course of the test development. With regard to the results of the factor-analytical evaluations, a bifactor model with a central factor “mental pain” and the five domain-specific factors “Somatization,” “Depression,” “Anxiety,” “Intrusive Memories,” and “Dissociation” could be established. The originally assumed dimensions “Compensation Behavior” and “Vulnerability” could not be confirmed. The aspect of “treatment,” which is important for expert practice, is assessed descriptively in the questionnaire, but is not included in the calculation of the total score. In the final version of the questionnaire, this total value of “mental pain” is formed by adding up the five sub-scales to which six items are assigned.

Originally, 77 items were constructed with regard to theoretical considerations and observations from the qualitative content analysis. In consecutive steps, 43 items were eliminated based on the results of the item analysis and analysis of the factor structure. Despite this item reduction, the values of the internal consistency, which are in a consistently satisfactory range for the final version with 30 items, indicate a high level of reliability (Bühner, 2011). In order to determine the stability of the FESSTE, however, further studies to determine the retest reliability must be carried out.

The preliminary test version obtained through exploratory factor analysis with 52 items with five factors could not be confirmed in a subsequent confirmatory factor analysis. However, a further item reduction based on the item-total correlation analysis resulted in a 30-item version of the questionnaire with a satisfactory model fit. In a direct comparison between several conceivable models, the bifactor model proved to be the latent structure with the best fit with regard to the empirically obtained data. It can therefore be assumed that the FESSTE is made up of an overall scale of “mental pain” and five residual factors that can be interpreted as subordinate factors (Chen et al., 2006). In correspondence to this, one might understand the high intercorrelations between the individual sub-factors (Cohen, 1992), resembling the correlation pattern of the BSI sub scales (Franke et al., 2011), which according to more recent findings exhibits a latent bifactor structure, as well (Thomas, 2012; Urbán et al., 2014).

Furthermore, the results of this study indicate a substantial positive correlation between the FESSTE and the applied measures for post-traumatic stress disorder and general psychiatric symptoms. These results suggest a high degree of convergent validity (Bühner, 2011). In particular, the overlap between the global scale of the BSI-18 and the FESSTE is in an area that raises the question of a differentiation between the general psychiatric distress measured with the BSI and the concept of mental pain. It should be noted that there is an increasing trend in recent psychiatric literature which instead of a purely categorical separation between mental illnesses, recognizes both broad, unspecific factors and domain-specific factors with regard to the latent structure of psychiatric phenomena (Watson, 2005; Tackett et al., 2008; Goodkind et al., 2015). In correspondence to this, it can be assumed that the mental pain measured with the FESSTE has a strong conceptual overlap with the global symptom severity measured with the BSI. The further calculations with a hierarchical multiple regression show, however, that the FESSTE is superior to both the PTSS-10 and the BSI-10 in predicting the extent of traumatic experiences. Hence, the data obtained in this study suggested the conclusion that the FESSTE is a more suitable measuring instrument for expert work. To further investigate the validity of FESSTE, future studies could investigate connections with the Orbach and Mikulincer Mental Pain Scale (OMMP, Orbach et al., 2003b), as well as increased suicidality (see Shneidman, 1998).

The total score of the FESSTE shows no significant age effects and the FESSTE subscales also only show negligible correlations with age, if at all. On the other hand, there are slight to medium gender differences in the answers to the FESSTE, which suggest a higher degree of mental pain in women. This finding is in line with epidemiological studies that show a higher prevalence of internalizing disorders, such as depression and PTSD in women (Kendler et al., 2003; Kessler, 2003; Bangasser and Valentino, 2014), while men show a higher probability of externalizing disorders—such as addictions (Grant et al., 2015). The latter are not recorded in the current version of FESSTE. An originally created scale that would have recorded externalizing “compensatory behavior” was removed due to inadequate psychometric properties. However, since traumatic addiction development and other externalizing disorders play a role in the assessment of mental pain, albeit in rarer cases, an extension of the FESSTE in this regard could be useful.

The current version of FESSTE has a trauma checklist oriented toward the results of Perkonigg et al. (2000) and the recently published DSM-5 (American Psychiatric Association, 2013). This enables experts to make an economic and structured assessment of the extent of the experienced traumatic events. The results imply that the traumatization experienced was closely related to the self-assessed mental pain. However, new research results show that this connection is substantially mediated by the structural integrity of personality structure (Fuchshuber et al., 2019; Flenreiss-Frankl et al., 2021). The mental pain triggered by traumatization should therefore be understood within the framework of an interplay of traumatic events and resilience or vulnerability that vary between individuals. What is more, it has to be noted that the extent of traumatic experiences scale showed only low reliability in this study. Regarding the wide variety of possible traumatic events, this circumstance might be expected. While the scale might be useful for practitioners concerned with forensic opinions due to its descriptive value, researcher who employ this scale into their studies should be conscious of its limited internal consistency and hence, interpret its score with caution.

### Limitations and Research Perspectives

A limitation of the validation study of the FESSTE results from the imbalance of the gender ratio within the sample. Comparatively few men took part in the examinations, which prevents the questionnaire from being standardized according to gender. On a similar note, the investigated sample shows an imbalance toward a relatively high proportion of participants with high education levels, which further inhibits the standardization of the scale based on the general population. In order to establish more reliable norm values and investigate for factorial invariance of the latent factor structure, it is therefore crucial that future studies are undertaken which explore more representative population samples.

Moreover, the FESSTE, like comparable self-assessment procedures, is prone to deliberate falsification. The use of suitable control procedures for socially desirable response styles could represent a possible addition to FESSTE. However, these instruments are only of limited effectiveness (van de Mortel, 2008). Hence, expert instinct and experience seem to play a particularly important role in assessing mental pain.

Furthermore, the ability of FESSTE to assess the time of mental pain after trauma is very limited. For this purpose, the development of a standardized interview is planned, which is created based on the questionnaire and enables an assessment of the respective symptom groups using temporal categories.

In addition to the creation of a standardized interview based on the results of the FESSTE, the development of a short version of the FESSTE is planned, which could be suitable to further sharpen the psychometric properties and the economic validity of the FESSTE.

In this study, which was based on data of the general population, the FESSTE scales did not show a normal distribution. However, comparable measuring instruments like the BSI showed similar results (Gilbar and Ben-Zur, 2002; Spitzer et al., 2011). In contrast, a normal distribution of the FESSTE scales is to be expected in clinical populations. It is therefore necessary to investigate patient groups in future studies concerned with norm data of the FESSTE.

Finally, the results might be influenced by the mode of data collection. While recent evaluations of the validity of online surveys underscored that they are comparable to more traditional surveys (Wiersma, 2013), it is yet to be investigated if the results of the present study can be extended to the paper-pencil version of the FESSTE.

## Conclusion

The aim of the work was to develop a questionnaire able to assess mental pain after traumatic experiences. With the FESSTE, a corresponding instrument is now available which enables an operationalization of mental pain on the basis of five subscales and one total scale. What is more, the trauma checklist attached to the FESSTE allows for the standardized assessment of potentially traumatic experiences and the corresponding extent of these experiences. In particular, the questionnaire is—with the exception of the extent of traumatic event scale—characterized by very high internal consistencies regarding its subscales and overall scale. Moreover, the results of the confirmatory factor analysis confirm the satisfactory structural validity of the questionnaire construction. The strong associations with relevant external criteria—such as psychiatric symptoms and the extent of traumatic experiences—indicate the high criterion validity of this new procedure. Finally, the FESSTE can be used without high economic expense, which makes it a useful instrument for approaching mental pain after traumatic events. While the limitations of this study discussed above imply the need for further research regarding standardization and factorial invariance, in summary, the results of this first examination indicate that the FESSTE is a reliable and valid self-assessment procedure, suitable for the use in research and in expert practice.

## Data Availability Statement

The raw data supporting the conclusions of this article will be made available by the authors, without undue reservation.

## Ethics Statement

The studies involving human participants were reviewed and approved by Ethics board, University of Nicosia. The patients/participants provided their written informed consent to participate in this study.

## Author Contributions

KF-F, JF, and HU developed the study and conducted the data analysis. JF conducted the data collection. KF-F and JF wrote the first draft of the manuscript. HU proofread the manuscript and made some critical comments. All authors read the final version of the manuscript and gave their consent for publication.

## Conflict of Interest

The authors declare that the research was conducted in the absence of any commercial or financial relationships that could be construed as a potential conflict of interest.
