# Supplementary material for: The Development of a Multidimensional Inventory for the Assessment of Mental Pain (FESSTE 30)
Source: Front Psychol. 2021 Apr 28;12:656862. doi: 10.3389/fpsyg.2021.656862 (PMC8115402; doi:10.3389/fpsyg.2021.656862)
Supplement: Supplementary file 1 [file Table_1.DOCX]

**Supplementary materials:**

**Final Form of the Questionnaire for the Assessment of Mental Pain after Traumatic events (FESSTE-30; German version)**

Im Folgendem werden verschiedene Beschwerden und Probleme aufgelistet, die Menschen manchmal nach der Erfahrung von schwerwiegenden oder traumatischen Ereignissen in ihrem Leben haben. Bitte lesen Sie jede Aussage sorgfältig durch und entscheiden Sie, wie sehr diese innerhalb der letzten 4 Wochen auf Sie zutreffen. Auch wenn die Fragen sehr persönlich sind, versuchen Sie bitte, sie so ehrlich wie möglich zu beantworten.

| 1 | 2 | 3 | 4 | 5 |
| --- | --- | --- | --- | --- |
| überhaupt nicht | ein wenig | ziemlich | stark | sehr stark |

|  |  | **Trifft auf mich zu ...** | | | | |
| --- | --- | --- | --- | --- | --- | --- |
| 1. Ich habe Rückenschmerzen. | | 1 | 2 | 3 | 4 | 5 |
|  |  |  | | | | |
| 2. Ich habe Muskelschmerzen. | | 1 | 2 | 3 | 4 | 5 |
|  |  |  | | | | |
| 3. Ich habe Schwierigkeiten bei der Atmung. | | 1 | 2 | 3 | 4 | 5 |
|  |  |  | | | | |
| 4. Ich leide unter Schwächegefühlen in bestimmten Körperteilen. | | 1 | 2 | 3 | 4 | 5 |
|  |  |  | | | | |
| 5. Ich leide unter Kribbeln oder Taubheit in bestimmten Körperteilen. | | 1 | 2 | 3 | 4 | 5 |
|  |  |  | | | | |
| 6. Ich leide unter Hitzewallungen oder Kälteschauern. | | 1 | 2 | 3 | 4 | 5 |
|  |  |  | | | | |
| 7. Ich fühle mich schwermütig. | | 1 | 2 | 3 | 4 | 5 |
|  |  |  | | | | |
| 8. Ich habe das Gefühl nichts mehr wert zu sein. | | 1 | 2 | 3 | 4 | 5 |
|  |  |  | | | | |
| 9. Wenn ich an die Zukunft denke, fühle ich mich hoffnungslos. | | 1 | 2 | 3 | 4 | 5 |
|  |  |  | | | | |
| 10. Ich habe das Gefühl, keine Energie mehr zu haben. | | 1 | 2 | 3 | 4 | 5 |
|  |  |  | | | | |
| 11. Es fällt mir schwer, mich zu konzentrieren. | | 1 | 2 | 3 | 4 | 5 |
|  |  |  | | | | |
| 12. Ich habe das Gefühl, die Kontrolle über mein Leben verloren zu haben. | | 1 | 2 | 3 | 4 | 5 |
|  |  |  | | | | |
| 13. Mein Schlaf ist unruhig. | | 1 | 2 | 3 | 4 | 5 |
|  |  |  | | | | |
| 14. Ich habe wiederkehrende Albträume über das Ereignis. | | 1 | 2 | 3 | 4 | 5 |
|  |  |  | | | | |
| 15. Ich muss das Ereignis immer wieder durchleben. | | 1 | 2 | 3 | 4 | 5 |
|  |  |  | | | | |
| 16. Wenn ich mich an das Ereignis erinnere, treten körperliche Reaktionen bei mir auf (Schweißausbrüche, Zittern, usw.). | | 1 | 2 | 3 | 4 | 5 |
|  |  |  | | | | |
| 17. Ich muss immer wieder an das Ereignis denken, obwohl ich es nicht will. | | 1 | 2 | 3 | 4 | 5 |
|  |  |  | | | | |
| 18. Ich versuche, nicht an das Ereignis zu denken. | | 1 | 2 | 3 | 4 | 5 |
|  |  |  | | | | |
| 19. Ich habe Angst auf der Straße oder vor offenen Plätzen. | | 1 | 2 | 3 | 4 | 5 |
|  |  |  | | | | |
| 20. Ich fürchte mich davor, allein aus dem Haus zu gehen. | | 1 | 2 | 3 | 4 | 5 |
|  |  |  | | | | |
| 21. Ich erschrecke, obwohl es keinen Grund dafür gibt. | | 1 | 2 | 3 | 4 | 5 |
|  |  |  | | | | |
| 22. Ich habe Angst vor Fahrten mit dem Bus, Straßenbahn, U-Bahn oder Zug. | | 1 | 2 | 3 | 4 | 5 |
|  |  |  | | | | |
| 23. Ich leide unter Panikanfällen. | | 1 | 2 | 3 | 4 | 5 |
|  |  |  | | | | |
| 24. Ich fürchte mich vor Menschenmengen. | | 1 | 2 | 3 | 4 | 5 |
|  |  |  | | | | |
| 25. Es gibt Momente, in denen ich mich so fühle, als sei ich nicht Teil von dem, was passiert. | | 1 | 2 | 3 | 4 | 5 |
|  |  |  | | | | |
| 26. Mein Zeitgefühl hat sich verändert. | | 1 | 2 | 3 | 4 | 5 |
|  |  |  | | | | |
| 27. Die Realität scheint mir unwirklich, wie in einem Traum oder einem Film. | | 1 | 2 | 3 | 4 | 5 |
|  |  |  | | | | |
| 28. Es gibt Momente, in denen ich in den Spiegel schaue und mich nicht erkenne. | | 1 | 2 | 3 | 4 | 5 |
|  |  |  | | | | |
| 29. Ich habe das Gefühl, mein eigener Körper gehört nicht zu mir. | | 1 | 2 | 3 | 4 | 5 |
|  |  |  | | | | |
| 30. Es kommt mir so vor, als wäre ich gefühlstaub. | | 1 | 2 | 3 | 4 | 5 |
|  |  |  | | | | |

Wie sehr fühlten Sie sich im allgemeinen, **durch die auf Sie zutreffenden Symptome,** in den (ungefähr) ersten 4 Wochen, zwischen dem 2. und 4. Monat, zwischen dem 5. Monat und dem 2. Jahr und seit dem 3. Jahr nach dem Ereignis beeinträchtigt? Sollte weniger Zeit seit dem traumatischen Ereignis vergangen sein, lassen die betreffende(n) Zeile(n) frei.

|  | **Die Beeinträchtigung war…** | | | | |
| --- | --- | --- | --- | --- | --- |
| Ca. innerhalb des 1. Monats nach dem Ereignis | 1 | 2 | 3 | 4 | 5 |
| Ca. zwischen dem 2. und 4. Monat nach dem Ereignis | 1 | 2 | 3 | 4 | 5 |
| Ca. zwischen dem 5. Monat und 2. Jahr nach dem Ereignis | 1 | 2 | 3 | 4 | 5 |
| Ca. seit dem 3. Jahr nach dem Ereignis | 1 | 2 | 3 | 4 | 5 |

Bitte lesen Sie die folgenden Aussagen sorgfältig durch und entscheiden Sie, wie sehr diese auf Sie zutreffen.

|  |  | **Trifft auf mich zu ...** | |
| --- | --- | --- | --- |
| 31. Ich befinde mich in psychiatrischer oder psychologischer Behandlung aufgrund von Beschwerden, die im Zusammenhang mit dem Ereignis stehen. | | Ja  2  3 | Nein  5 |
|  |  |  |  |

|  |  |  | |
| --- | --- | --- | --- |
| 32. Mir wurden aufgrund psychischer Beschwerden, die im Zusammenhang mit dem Ereignis stehen, Medikamente verordnet. | | Ja  2  3 | Nein  5 |
|  |  |  |  |

|  |  |  | |
| --- | --- | --- | --- |
| 33. Ich befinde mich aufgrund psychischer Beschwerden, die im Zusammenhang mit dem Ereignis stehen, in Behandlung. | | Ja  2  3 | Nein  5 |
|  |  |  |  |
|  |  |  | |
| 34. Bereits vor dem Ereignis war ich aufgrund psychischer Probleme in medizinischer oder psychologischer Behandlung. | | Ja  2  3 | Nein  5 |
|  |  |  |  |

|  |  |  | |
| --- | --- | --- | --- |
| 35. Mir wurden aufgrund psychischer Beschwerden, die im Zusammenhang mit dem Ereignis stehen, Medikamente verordnet. | | Ja  2  3 | Nein  5 |
|  |  |  |  |

|  |  |  | |
| --- | --- | --- | --- |
| 36. Ich befinde mich aufgrund psychischer Beschwerden, die im Zusammenhang mit dem Ereignis stehen, in Behandlung. | | Ja  2  3 | Nein  5 |
|  |  |  |  |
|  |  |  | |
| 37. Bereits vor dem Ereignis war ich aufgrund psychischer Probleme in medizinischer oder psychologischer Behandlung. | | Ja  2  3 | Nein  5 |
|  |  |  |  |
|  |  |  | |
| 38. Bereits vor dem Ereignis wurde bei mir mindestens eine psychische Störung diagnostiziert. | | Ja  2  3 | Nein  5 |
|  |  |  |  |

39. Wenn ja, welche Störung/Störungen wurden bei Ihnen diagnostiziert?

Bitte hier beantworten:

___________________________________________________________________________

___________________________________________________________________________

40. Leiden Sie unter zusätzlichen Beschwerden, die im Zusammenhang mit dem Ereignis stehen, die im Fragebogen nicht genannt wurden?

Bitte hier beantworten: _______________________________________________________________________________________________________________________________________________________________________________________________________________________________________________________________________________________________________________________________________________________________________________________

## **Traumachecklist**

Im Folgenden sind eine Reihe von schwerwiegenden oder traumatischen Lebensereignissen aufgelistet, wie z. B. Erfahrungen mit körperlichem oder seelischem Missbrauch, Katastrophen oder Krieg. Geben sie bitte für jedes Ereignis an, ob Sie es erlebt haben, und wenn ja, ob es Ihnen persönlich passierte oder Sie Zeuge dieses Ereignisses wurden.

|  | Nein | Ja | |
| --- | --- | --- | --- |
|  |  | Persön-lich | Als Zeuge |
| 1. Folter |  |  |  |
| 2. Aufenthalt in Kriegsgebiet |  |  |  |
| 3. Schwerer Unfall, Feuer oder Explosion |  |  |  |
| 4. Naturkatastrophe |  |  |  |
| 5.Schwere Krankheit oder Verletzung |  |  |  |
| 6. Plötzlicher oder unerwarteter Tod eines nahen Angehörigen oder einer wichtigen Bezugsperson |  |  |  |
| 7. Flucht und Migration |  |  |  |
| 8. Gefangenschaft |  |  |  |
| 9. Vernachlässigung oder Verwahrlosung |  |  |  |
| 10. Sexuelle Gewalt durch eine fremde Person als Erwachsener |  |  |  |
| 11. Sexuelle Gewalt durch eine Person aus dem Familien- oder Bekanntenkreis |  |  |  |
| 12. Sexuelle Gewalt als Kind oder Jugendlicher durch eine fremde Person |  |  |  |
| 13. Sexuelle Gewalt als Kind oder Jugendlicher durch eine Person aus dem Familien- oder Bekanntenkreis |  |  |  |
| 14. Gewalttätiger Angriff durch eine fremde Person |  |  |  |
| 15. Gewalttätiger Angriff durch eine Person aus dem Familien- oder Bekanntenkreis |  |  |  |
| 16a. Andere traumatische Ereignisse |  |  |  |
|  | | | |

16b. Wenn Sie 16a. mit Ja beantwortet haben, beschreiben Sie bitte dieses Ereignis oder diese Ereignisse in kurzen Stichpunkten: ____________________________________________________________________________________________________________________________________________________________________________________________________________________________________________________________________________________________________________

17. Welches der traumatischen Ereignisse, die Sie mit „Ja, persönlich“ oder „Ja, als Zeuge“ beantwortet haben, war für Sie das schlimmste Ereignis?

(1-16)

18. Wie viel Zeit ist seit dem schlimmsten Erlebnis ungefähr vergangen?

Wochen:

Monate:

Jahre:
